# Supplementary material for: The interactome of intact mitochondria by cross-linking mass spectrometry provides evidence for coexisting respiratory supercomplexes
Source: Mol Cell Proteomics. 2017 Dec 8;17(2):216–32. doi: 10.1074/mcp.RA117.000470 (PMC5795388; doi:10.1074/mcp.RA117.000470)
Supplement: Supplemental Data [file supp_RA117.000470_133922_0_supp_23978_fzffwf.zip › spectra_annotation/mito_spectra_annotation/383-1-1-1-18-1.pdf]

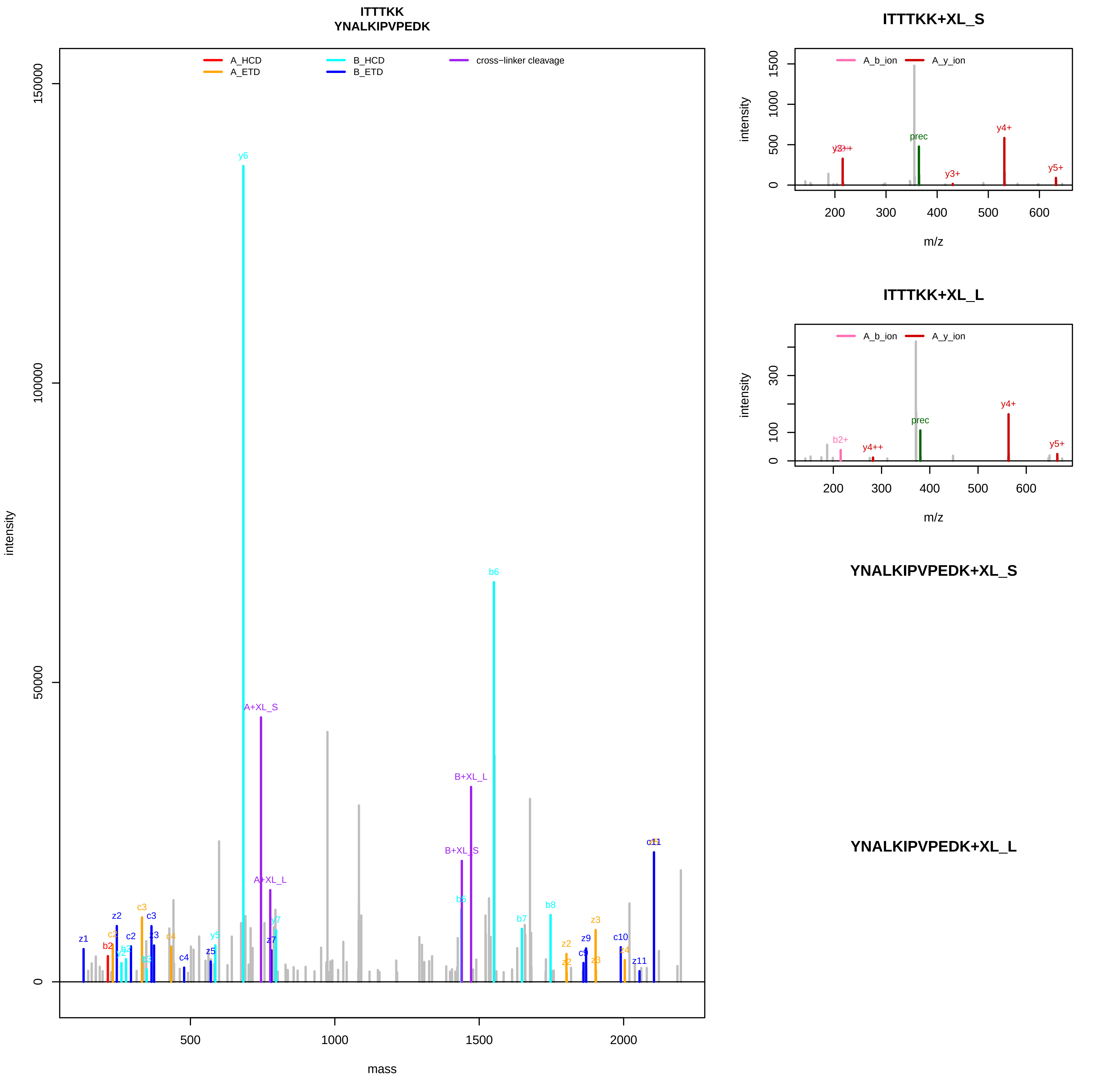
ITTTKK
YNALKIPVPEDK
A_HCD
A_ETD
B_HCD
B_ETD
cross−linker cleavage
intensity
mass
150000
100000
50000
0
500
1000
1500
2000
y6
b6
A+XL_S
A+XL_L
B+XL_L
B+XL_S
b5
b7
b8
c11
z1
z2
c2
b2
c3
z3
c4
z5
y5
y7
z7
z9
c10
z11
z2
z3
c4
ITTTKK+XL_S
A_b_ion
A_y_ion
y3++
prec
y4+
y3+
y5+
m/z
ITTTKK+XL_L
b2+
y4++
YNALKIPVPEDK+XL_S
YNALKIPVPEDK+XL_L
